# Small-angle scattering and morphologies of ultra-flexible microemulsions^1^

**DOI:** 10.1107/S1600576716016150

**Published:** 2016-11-11

**Authors:** Sylvain Prevost, Tobias Lopian, Maximilian Pleines, Olivier Diat, Thomas Zemb

**Affiliations:** a ESRF – The European Synchrotron, 71 avenue des Martyrs, 38000 Grenoble, France; b Institut de Chimie Séparative de Marcoule (ICSM), UMR 5257 (CEA/CNRS/UM2/ENCSM), 30207 Bagnols-sur-Cèze, France; c Institute of Physical and Theoretical Chemistry, University of Regensburg, 93040 Regensburg, Germany

**Keywords:** ultra-flexible microemulsions, surfactant-free microemulsions, mesoscale solubilization, small-angle scattering (SAS), ternary phase diagrams

## Abstract

The phase diagrams of ternary mixtures of partly miscible solvents containing a hydrotropic co-solvent exhibit a variable miscibility gap and one critical point. This work investigates the entire monophasic region far from and near to the miscibility gap in octan-1-ol/ethanol/water, for which ultra-flexible micro­emulsions (UFMEs) are observed by small-angle scattering techniques. SWAXS (combined small- and wide-angle X-ray scattering) allows the elucidation of these types of structure. Three distinct areas can be identified in the phase diagram, with scattering data resembling those from direct, bicontinuous and reverse local structures. These UFMEs are far more polydisperse than their surfactant-based counterparts. Water-rich and solvent-rich domains are only delimited by a small excess of hydrotrope, instead of a well defined surfactant layer of fixed area per molecule. It is shown that all scattering spectra obtained for the nanostructured compositions can be modelled by a simple unified analytical model composed of two uncorrelated contributions. The main one is the Ornstein–Zernike formula for composition fluctuations which gives information about the pseudo-phase domain size. The second is a Lorentzian that captures the structure of at least one of the coexisting pseudo-phases. No Porod law can be measured in the SAXS domain. The proposed expression gives access to two characteristic sizes as well as one inter-aggregate distance.

## Abstract 2

Three-component fluids can exhibit structured density fluctuations, and their small-angle scattering patterns present similarities to those of classical microemulsions. One general analytical expression with two additive contributions (one for the two immiscible fluids and a smaller one for a structured fluid) allows the whole phase diagram to be mapped in the single-phase domain.

## 1. Introduction   

Classical microemulsions are thermodynamically stable mixtures of two immiscible fluids whose domains are spatially isolated and stabilized by a monomolecular surfactant film. Ultra-flexible microemulsions (UFMEs) achieve the same thermodynamic stability with two mutually immiscible fluids and the addition of a hydrotropic co-solvent (usually a short-chain alcohol) rather than a surfactant. On the water-rich side of the triangular phase diagram and near the phase boundary, these UFMEs have been discovered as a form of ‘unexplained solution’. Under moderate ultracentrifugation conditions, they exhibit three phases instead of the two seen for classical centrifugation procedures, due to creaming or sedimentation, discovered by Svedberg in his Nobel Prize work (Svedberg & Rinde, 1924 ▸). They were named ‘detergentless microemulsions’ by Barden and co-workers (Smith *et al.*, 1977 ▸) in the 1970s and were referred to as mesoscale inhomogeneities by Anisimov and coworkers (Robertson *et al.*, 2016 ▸). We have named them ‘ultra-flexible microemulsions’, to distinguish them from classical flexible or rigid microemulsions where curvature plays a role in free energy (Zemb *et al.*, 2016 ▸). In these types of microemulsion, polar and apolar domains are also spatially separated. However, the hydrotrope solubilizes in both domains and a slight accumulation at a diffuse interface has been proven (Schöttl *et al.*, 2014 ▸). This enrichment increases the ‘hydration force’ (Donaldson *et al.*, 2015 ▸) that competes with entropy and thus allows a dispersion of one solvent in the other (Zemb *et al.*, 2016 ▸). This competition controls the average size of the aggregates, which feature a broad size distribution.

Practically, being able to mix solvents possessing completely different solubilization parameters and obtain a clear monophasic fluid combining properties of its components while requiring no classical surfactant/detergent has important consequences. For extraction such as in the case of selective recycling, amphiphilic extraction molecules are poorly volatile and thus difficult to remove by evaporation, but they are also too small to remove by filtration. For catalysis, surfactants create a membrane around droplets that may be difficult to penetrate. Generally, surfactants may interact chemically with additives, or deteriorate with oxygen, light, or alkaline or acidic conditions. These ternary mixtures also have interesting non-ideal behaviour for the activity of their components, a fact already exploited for a long time, for example, in the manufacture of fragrances (Marcus *et al.*, 2015 ▸). Ternary fluids have been heavily used in industry ever since the formulation of ‘Eau de Cologne’ in the early eighteenth century, for the co-extraction of flavours and fragrances of very different hydrophobicities.

Qualitative microstructural studies of ternary systems are relatively recent in the literature and have focused on the existence and structure of clusters, combining scattering techniques, such as dynamic and static light scattering and small-angle scattering (SAS) (Klossek *et al.*, 2012 ▸; Diat *et al.*, 2013 ▸), with simulations (Schöttl *et al.*, 2014 ▸; Schöttl & Horinek, 2016 ▸; Lopian *et al.*, 2016 ▸).

From previous studies, it is known that water-rich and solvent-rich domains coexist. The chemical equilibrium of the hydrotropic co-solvent determines the stability of the meso­scopic phase separation. The morphological domains are conveniently modelled by Gaussian random fields, with a single correlation length larger than a molecular length (Arleth *et al.*, 2001 ▸). Moreover, it is known that both the water-rich and solvent-rich domains are structured, with a smaller correlation length, where fluctuations in the domain sizes are associated with a gain in molecular entropy and a loss in solvation entropy (Kunz *et al.*, 2016 ▸).

For decades, the complexity of apparently simple fluids has been questioned, and as early as 26 years ago the scattering from binary mixtures of heavy water and fully water-miscible short-chain alcohols was analysed in terms of Ornstein–Zernike (OZ) fluctuations by D’Arrigo & Teixeira (1990 ▸). These authors proposed the existence of ‘micelle-like’ structures of alcohol or water–alcohol complexes. It was recently found that binary mixtures of alcohol and toluene or cyclohexane (Mhanna *et al.*, 2016 ▸) and of alcohols and ionic liquids (Russina *et al.*, 2014 ▸; Schroer *et al.*, 2016 ▸; Murphy *et al.*, 2016 ▸) also exhibit density fluctuations, with clusters and bi­continuous alcohol-rich domains. Kononov recently highlighted the existence and probable role of nanometric concentration fluctuation in liquids (Kononov, 2015 ▸) to explain chemical reactivity not simply from a molecular point of view but taking into account these fluctuations for their consequences on the activity of the components, thus justifying the need for a ‘paradigm shift’. Finally, there has been a resurgence of work on the structure of water at room temperature, showing, all by itself, OZ fluctuations, even well above its melting point and away from any remarkable temperature and pressure point, with on-going discussions as to the origin of such fluctuations (Bosio *et al.*, 1981 ▸; Huang *et al.*, 2009 ▸, 2010 ▸; Skinner *et al.*, 2013 ▸; Sedlmeier *et al.*, 2011 ▸; Clark *et al.*, 2010 ▸).

Hence, density fluctuations in multicomponent fluids that show sometimes surprisingly intense scattering spectra – without micelle-forming surfactants, detergents or lipids – have become a popular subject. Beyond parameter fitting, predictive models can be either proved or disproved by high-quality small- and wide-angle scattering data that cover the appropriate spatial window (molecular to nanometre scale). In this paper, we propose a general formalism for these structures, whose scattering signals exhibit no Porod-limit behaviour (Gradzielski *et al.*, 1996 ▸) and do not always follow the extended OZ behaviour that has been proposed near a critical point in the presence of an impurity (Onuki & Kitamura, 2004 ▸; Bier & Harnau, 2012 ▸; Witala *et al.*, 2016 ▸).

We investigate these questions in detail for the ternary model system octan-1-ol/ethanol/water throughout the mono­phasic region, using combined small- and wide-angle X-ray scattering (SWAXS) performed on beamline ID02 at the ESRF.

## 2. Material and methods   

### 2.1. Materials   

Octan-1-ol (≥98%) and ethanol (≥99.8%) were purchased from Sigma–Aldrich and used as received. Ultrapure water (18.2 MΩ) was produced from a Millipore Milli-Q system.

### 2.2. Sample preparation   

Typically, the oil (octan-1-ol) and hydrotrope (ethanol) were mixed in the desired proportion, then water was added until turbidity was observed. Then, the oil/hydrotrope mixture was added dropwise until the sample became clear again. Since the samples are at equilibrium, it is possible to prepare them differently, *e.g.* starting with the biphasic oil/water and adding the hydrotrope until a clear monophasic solution is obtained. Note, however, that we do observe hysteresis, indicating that, in the vicinity of the miscibility gap, some time is needed for full equilibration. All additions were measured by weight with a laboratory scale. Solutions were prepared at room temperature [297 (1) K].

### 2.3. Methods   

SWAXS data were acquired on beamline ID02 at the ESRF on several occasions. A wavelength of 12.46 keV (λ = 0.0995 nm) was chosen. Two CCD detectors from Rayonix were used (MX170-HS and LX170-HS, respectively, for SAXS and WAXS), with a geometry allowing simultaneous acquisition with an overlap in *Q*, the magnitude of the wavevector [*Q* = 4π/λsin(θ/2), θ being the scattering angle]. The sample-to-detector distances were approximately 1.5 m for the SAXS detector and 0.13 m for the WAXS, calibrated by the Bragg peaks of *para*-bromobenzoic acid (SAXS and WAXS), of silver behenate (SAXS) and of silicon powder (WAXS). Samples were inserted into a thermalized (298 K) flow-through quartz capillary of inner diameter around 1.7 mm, as measured by transmission scan. Data were corrected for the dark current, the flat field, the incoming flux and the transmitted beam; note that effects from the beam polarization and the sample geometry were not taken into account during data reduction. An absolute scale was obtained using the plateau intensity level of water (at 1.63 × 10^−3^ mm^−1^ at 298 K). The combined SAXS and WAXS setup with overlap requires an air gap to be left of *ca* 15 cm that produces significant background from air scattering, but this is mostly removed when subtracting the scattering from the empty capillary. At low *Q*, surface scattering from the quartz capillary interfaces becomes non-negligible, and such scattering depends on the contrast at the interface, *i.e.* the filling of the capillary, which is why below *Q* ≃ 0.2 nm^−1^ the data may present a downturn or, more rarely, an upturn. This can be accounted for in the fits by adding an α*Q* ^β^ contribution. The data were analysed with the fitting programs *SASET* (Muthig *et al.*, 2013 ▸) and *SASfit* (Breßler *et al.*, 2015 ▸).

## 3. Results   

### 3.1. SWAXS spectra of dilution lines   

**Figure 1:**
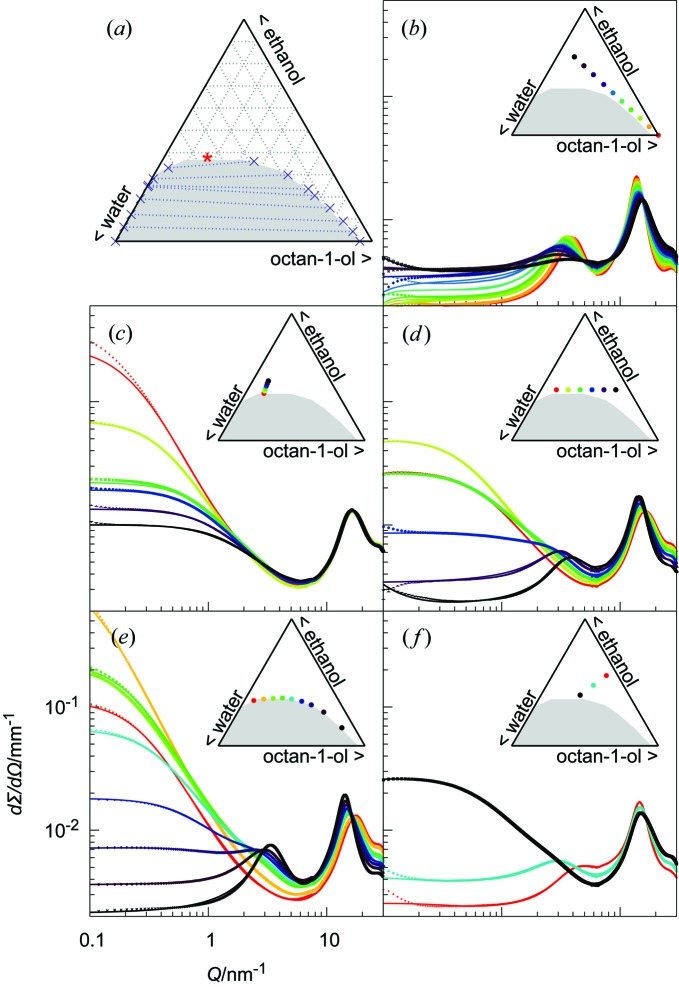
(*a*) Ternary phase diagram of octan-1-ol/ethanol/water by weight, showing the miscibility gap and the tie lines determined by Arce *et al.* (1994 ▸), and with the plait (critical) point located as a red star. (*b*)–(*f*) SWAXS spectra measured on ID02 at ESRF (points) for ternary mixtures of octan-1-ol/ethanol/water with the compositions shown on the ternary diagrams (insets). Continuous lines are fits according to equation (1). All spectra are at the same scale.

The subject of our study is the monophasic region of the well characterized model system octan-1-ol/ethanol/water. We chose this system as a reference, since all its chemical and physical properties are available (Arce *et al.*, 1993 ▸, 1994 ▸); octan-1-ol/water is the classical liquid–liquid equilibrium system to classify compounds based on their hydrophobicity (‘log*P*’), while ethanol is the most common hydrotrope used in industry (Kunz *et al.*, 2016 ▸). The phase diagram in mass fractions is shown in Fig. 1 ▸(*a*), indicating the miscibility gap of water and octan-1-ol and the monophasic region, obtained upon sufficient addition of ethanol. Water is slightly soluble in octan-1-ol, while octan-1-ol is much less soluble in water. The ternary phase diagram is also plotted in volume and mole fractions in the supporting information (Fig. S1). The tie lines of the ternary phase diagram in Fig. 1 ▸(*a*) indicate the distribution of ethanol between the coexisting water-rich and octan-1-ol-rich macroscopic phases: in mass and volume fractions, they lie roughly parallel to the water/octan-1-ol axis, implying that ethanol is essentially shared equally between the two macroscopic phases. Looking more precisely and considering the ternary diagram in mass fraction, there is a ‘rotation’: at ethanol concentrations below 25% in mass, ethanol will dissolve slightly better in octan-1-ol (ethanol is slightly ‘hydrophobic’), while for ethanol concentrations above 25%, the hydrotrope distributes preferentially towards the aqueous phase which becomes a ‘good solvent’ for the supplementary ethanol molecules (ethanol is slightly ‘hydrophilic’). Com­bined SWAXS measured on an absolute intensity scale is a unique tool for probing structural transitions in UFMEs, since it yields simultaneously quantitative information from the atomic scale (high *Q* values) up to the nano- and meso­scopic scales (low *Q* values). In previous work, investigations of microstructure in ternary mixtures focused on the so-called ‘pre-Ouzo’ region in the water-rich part of the phase diagram (Diat *et al.*, 2013 ▸) or on the formation of aggregates around the phase transition (Lopian *et al.*, 2016 ▸). The current work aims to cover the entire monophasic region of the phase diagram, and to do so several dilution lines are explored. The pathways chosen are illustrated in Figs. 1 ▸(*b*)–1 ▸(*f*) with the corresponding SWAXS spectra.

Fig. 1 ▸(*b*) shows spectra for a dilution line starting from pure octan-1-ol, with successive additions of a binary mixture of ethanol/water (70:30 by weight). All scattering spectra show the characteristic peak of octan-1-ol around 3–4 nm^−1^, a signal close to what is observed for classical reverse micelles. This peak exists in pure octan-1-ol and vanishes with increasing water/ethanol addition, while moving towards lower *Q*. The scattering intensity at low angles increases slowly throughout the series but stays within the range of a ternary molecular fluid that does not contain significant aggregates.

Fig. 1 ▸(*c*): initially, ethanol is added to a biphasic binary mixture of octan-1-ol and water (20:80) until a monophasic transparent solution is reached. This ternary mixture is located very near the plait (critical) point; with slightly less ethanol, a phase transition occurs (the ‘Ouzo’ region). Upon reaching the monophasic region, more ethanol is added and a spectrum is taken every 1% of further added hydrotrope. Along this path, we observe the development of the scattering spectra in the so-called pre-Ouzo region and its evolution the further away we move from the phase-transition border. In this series, no peak is seen at intermediate *Q*, but a large OZ signal is measured at low *Q* that decreases when moving away from the binodal.

Fig. 1 ▸(*d*): the dilution line is between two binary solutions of hydrotrope/solvent with a fixed composition of 40% in ethanol. Along this dilution line, the system passes just above the critical point and allows the study of the water–oil inversion. On the octan-1-ol-rich side, the spectra resemble those from Fig. 1 ▸(*b*), with a peak at intermediate *Q*; on the water-rich side, the spectra resemble those from Fig. 1 ▸(*c*), with a large signal at low *Q*. This cut at constant hydrotrope concentration illustrates the transition between morphologies.

Fig. 1 ▸(*e*): the points along the two-phase region are investigated. Those points were obtained in practice by preparing biphasic solutions of known octan-1-ol/water mass ratios and titrating with ethanol until the solutions become transparent. They are close to phase separation, and correspond to a standard formulation in fragrance and perfume formulation (Marcus *et al.*, 2013 ▸; Tchakalova *et al.*, 2014 ▸). The spectra are similar to those of Fig. 1 ▸(*d*), fully in the monophasic region and at constant ethanol mass fraction. With a few percent less ethanol present, the sample at highest water content, with a red spectrum, emulsifies spontaneously, while the ones on the right-hand side of the critical point will quickly phase separate. The one in yellow is close to the critical point and shows opalescence.

Fig. 1 ▸(*f*): starting from octan-1-ol/ethanol (40:60 by weight), water is added progressively. When using classical microemulsions, this type of dilution is called ‘water titration’, and it led to the discovery of the bicontinuous sponge phase that was described in papers by Fontell (1975 ▸) before being modelled by a simple expression reminiscent of a lamellar phase (Cabos *et al.*, 1988 ▸; Porte *et al.*, 1989 ▸; Gazeau *et al.*, 1989 ▸; Strey *et al.*, 1990 ▸). The signal increases at low *Q*, while a peak disappears at intermediate *Q*.

### 3.2. Scattering patterns   

**Figure 2:**
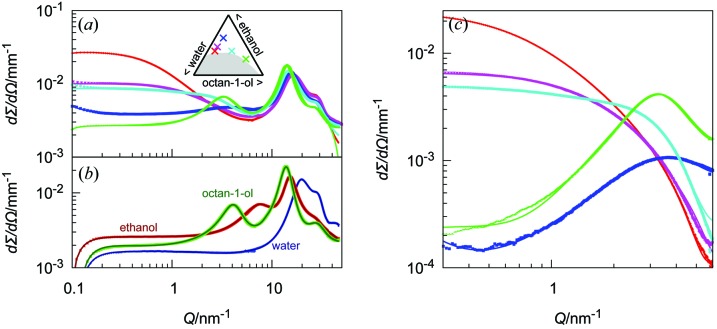
SWAXS spectra (ID02, ESRF) (*a*) of some characteristic ternary mixtures with compositions illustrated in the inset and (*b*) for the pure solvents octan-1-ol, ethanol and water. (*c*) The low-*Q* parts of the spectra in panel (*a*), plotted after background subtraction to highlight the different shapes of the curves,

Regarding the SWAXS spectra in Fig. 1 ▸, three *Q* ranges can be identified with different intensity behaviour. A few example spectra are replotted in Fig. 2 ▸, together with spectra from the three pure solvents. (i) At high *Q* values, above 7 nm^−1^ (2π/*Q* < 1 nm), interatomic scattering is observed, with two dominant peaks for correlations between C—O and C—C (intramolecular) and O⋯O (intermolecular). Both C—C and C—O bonds show scattering peaks at around 14 nm^−1^, while the O⋯O interactions show a scattering peak at around 28 nm^−1^. (ii) At intermediate *Q*, between 1 and 7 nm^−1^, a broad peak might be observed [see the green spectra in Fig. 2 ▸(*b*) for pure octan-1-ol and in Fig. 2 ▸(*a*) for the octan-1-ol-rich sample], which originates from correlations between pools of hydroxy groups, already present in pure linear alcohols (Tomšič *et al.*, 2007 ▸); the clusters of hydroxy groups in alcohols have been the focus of many articles, in particular from simulations and scattering [see, for example, Vahvaselkä *et al.* (1995 ▸) and Franks & Ives (1966 ▸)]. For pure octan-1-ol, the peak is at 4.1 nm^−1^. (iii) At low *Q*, below 1 nm^−1^, the intensity might decay, reach a plateau, or increase by orders of magnitude, the rising intensity revealing the existence of ‘domains’ of several nanometres. In the following, we will name these domains ‘water-rich’ near the water corner and ‘octan-1-ol-rich’ near the octan-1-ol corner of the phase diagram.

In the low-*Q* range, *i.e.* below a few reciprocal nanometres, we can group the spectra into four categories, from water-rich to octan-1-ol-rich compositions:

(i) A high intensity at low *Q* values, decaying as *Q* ^−*n*^, as determined by fits of a background and power law and no obvious peak in the intermediate-*Q* regime (red and purple spectra in Fig. 2 ▸ *a*).

(ii) A rather flat intensity at low *Q*, reaching a kink in the mid-*Q* regime (cyan spectrum).

(iii) Little intensity at low *Q*, with the characteristic correlation peak at intermediate *Q* values (green spectrum).

(iv) The fourth category is found at high ethanol concentrations. Here, we find a molecular solution, which is represented by a spectrum with no particular scattering pattern in the small-angle region (blue spectrum).

The spectra in categories (i)–(iii) can be directly related to microemulsions:

(i) Non-interacting large droplets that produce a large intensity upturn, reaching a plateau at low *Q* values, modelled by an analytical form factor.

(ii) Bicontinuous microemulsions that reach an intensity plateau at low *Q* values, below a peak or kink at intermediate *Q* corresponding to the distance between droplets; modelled with the Teubner–Strey expression which is able to fit any peak for systems showing a Porod limit, since the quadratic term is present (Teubner & Strey, 1987 ▸; Schubert *et al.*, 1994 ▸).

(iii) Concentrated microemulsions with a high number density of small droplets (either connected by coalescence or not) that essentially produce a peak with low intensity at low *Q*, which means a structure factor is visible at first glance in the scattering pattern, dominating any form factor.

By analogy between UFMEs and classical flexible or rigid microemulsions (Duvail *et al.*, 2013 ▸), we propose the following working hypothesis:

(*a*) The water-rich samples have similarities with oil–water microemulsions, including those close to the critical point. These show a fluctuation not in molecular concentration but in molecular aggregate concentration (Zemb *et al.*, 1990 ▸). The critical point is a critical point not of the molecular fluid but of aggregates fluctuating in concentration, as discovered by Corti & Degiorgio (1981 ▸).

(*b*) Octan-1-ol-rich samples behave like water–oil microemulsions (referred to as ‘reverse micelles’ for historical reasons; Zulauf & Eicke, 1979 ▸).

(*c*) In the intermediate region, the samples behave like ‘bicontinuous’ microemulsions, with a characteristic and unusual spectrum essentially flat at low *Q*.

### 3.3. Data modelling   

Classically, SAS data modelling relies on geometric and topological assumptions, the most basic being that the surface-to-volume ratio must be set by the concentration of surfactant present. Unfortunately, some published data do not check this requirement of the ‘best fit’, corresponding to a possible configuration assumed and tested *versus* the scattering signal measured (Barnes & Zemb, 1988 ▸; Milner *et al.*, 1988 ▸; Welberry & Zemb, 1988 ▸; Zemb & Welberry, 1993 ▸; Zemb, 2009 ▸). For homogeneous scatterers with a ‘sharp’ interface, a Porod law is always observed, *i.e.* the intensity at high *Q* decreases as a *Q* ^−4^ law, scaled in proportion to the contrast and specific amount of interface. This law is applicable for all types of emulsion and microemulsion, provided the interface is sharp at the resolution of the experiment (Lindner & Zemb, 2002 ▸). This Porod law behaviour was observed and used to unravel the processes occurring during ageing in the case of ‘Ouzo’ droplets (Grillo, 2003 ▸). In the present case, making all possible experiments around the two-phase region, the observed fluctuations never showed this *Q* ^−4^ trend in the intermediate range (though this decay might exist in a high-*Q* range dominated by molecular scattering). In order to reproduce the observed spectra from SAS on UFMEs, the following requirements were set for an appropriate model:

(i) It must provide a characteristic size of the scatterers in the nanometre domain.

(ii) It may or may not produce a peak. If such a feature is observed, a characteristic distance between the electron-dense heterogeneities should be provided.

(iii) A decay slower than the Porod law is necessary in the intermediate-*Q* regime.

(iv) The function has few features in general (in particular, none of the oscillations obtained from well defined geometric models), consistent with the relatively small amount of information in the scattering data.

**Figure 3:**
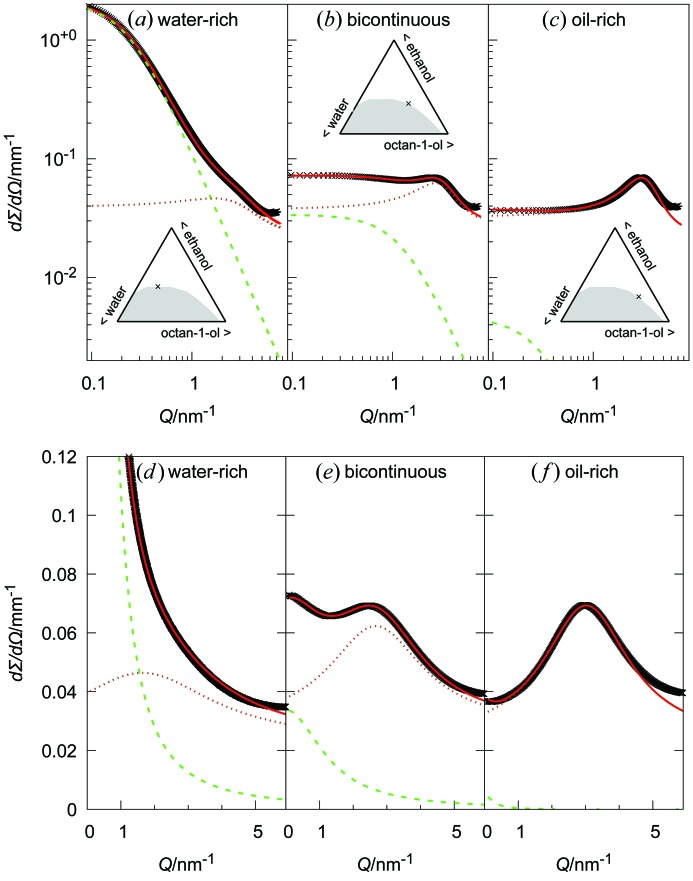
Experimental SWAXS spectra (ID02, ESRF) of three samples near the binodal, at mass fractions in octan-1-ol/ethanol/water of (*a*), (*d*) 0.188/0.373/0.439 for the water-rich sample, (*b*), (*e*) 0.4735/0.3227/0.2038 for the bicontinuous sample and (*c*), (*f*) 0.5864/0.2657/0.1479 for the oil-rich sample. Solid red lines are best fits to the two-contribution model [equation (1)], dashed green lines are the OZ contributions, and dotted dark-orange lines are the Lorentzian functions for dense clusters of hydroxy groups. Plots (*a*)–(*c*) are on a double logarithmic scale and plots (*d*)–(*f*) are on a double linear scale. The deviation at high *Q* can be avoided if the constant background used in these plots is replaced by the sum of the Lorentzian functions fitting the WAXS peaks (see *e.g.* the fits in Fig. 1 ▸). Similar plots for samples further away from the binodal are shown in Fig. S5 in the supporting information.

Figs. S2 to S4 in the supporting information show that different models could fit some of the spectra equally well, in particular in the intermediate regime where the Teubner–Strey model and the extended OZ model can reproduce the main experimental features. However, we find that the simple summation of OZ fluctuations and a broad-peak model, *i.e.* the sum of two Lorentzian functions, one with its mode at *Q* = 0 and one with its mode at an intermediate *Q* value, can reproduce all spectra in the SAXS *Q* range and only requires a minimum number of parameters: 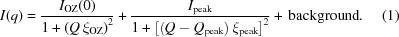
The scattering at low wavevector, due to the presence of ‘large’ aggregates, is reproduced *via* OZ with two parameters: the intensity scaling *I*_OZ_(0) and the characteristic size ξ_OZ_, an analysis done numerous times before by us and others, *e.g.* by D’Arrigo & Teixeira (1990 ▸) and Marcus *et al.* (2015 ▸). The peak sometimes observed at intermediate *Q* is reproduced *via* a Lorentzian with three parameters: the scale *I*_peak_, the peak position *Q*_peak_ corresponding to a characteristic distance 2π/*Q*_peak_, and the peak half-width at half-maximum 1/ξ_peak_ where ξ is, as in the OZ term, the spatial extent of the scatterer size. The background is either a constant, when considering purely the SAXS regime, as done in Fig. 3 ▸, or a sum of Lorentzian functions reproducing the peaks in the WAXS range, whose tails at low *Q* are rather flat, hence leading to an almost constant background, as done in Figs. 1 ▸ and 2 ▸ [*cf.* Figs. S5(*c*) and S6 in the supporting information].

### 3.4. Physical meaning of the formula   

**Figure 4:**
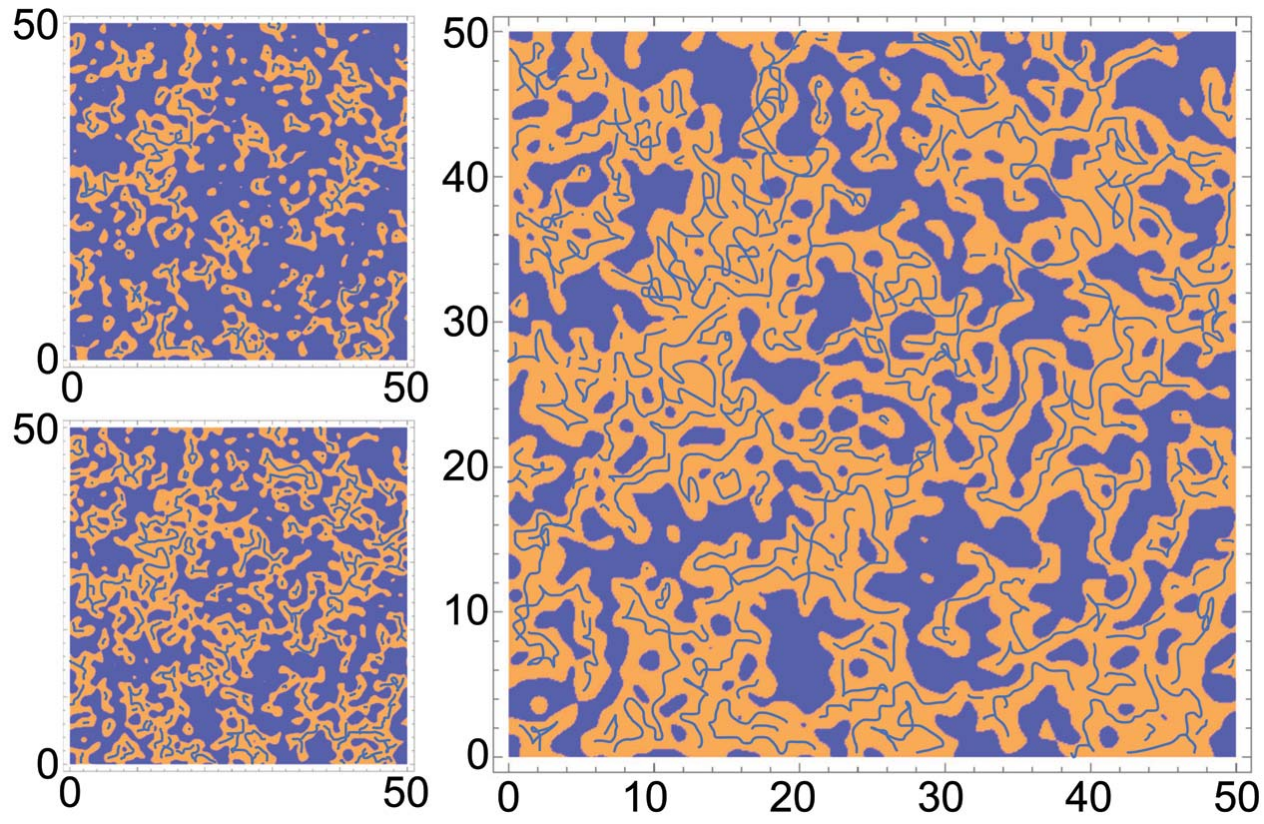
Schematic representations of the fluctuations between octan-1-ol-rich domains (orange) and water-rich domains (blue), with scales in nanometres. (Top left) 35% octan-1-ol. (Bottom left) 50% octan-1-ol. (Right) 65% octan-1-ol. The important length for the water-rich domains is the universal length λ = 0.27 nm (Parsegian & Zemb, 2011 ▸), while the octan-1-ol-rich part is a structured solvent mixture with a three-dimensional dynamic network of hydrogen bonds, created by alcohol groups and water (blue mesh in the orange domains), the mesh size of which is given by the peak position (2π/*Q*_peak_).

Schematically, in this formula we consider two domains of uncorrelated microstructures: water-rich regions on one side and, on the other, oil-rich regions that contain an additional structuring level with an orientation of molecules resulting in polar domains of a molecular size, as illustrated in Fig. 4 ▸. Here, polar and apolar domains are spatially separated, appearing as clusters. The size of such a cluster is the length ξ_OZ_, but as depicted the size distribution is broad. On the other hand, the polar hydroxy groups of octan-1-ol are not distributed randomly, but orient towards each other and form a loose network inside the apolar domain, shown as a dark-blue web in Fig. 4 ▸.

### 3.5. Results of the fits   

**Figure 5:**
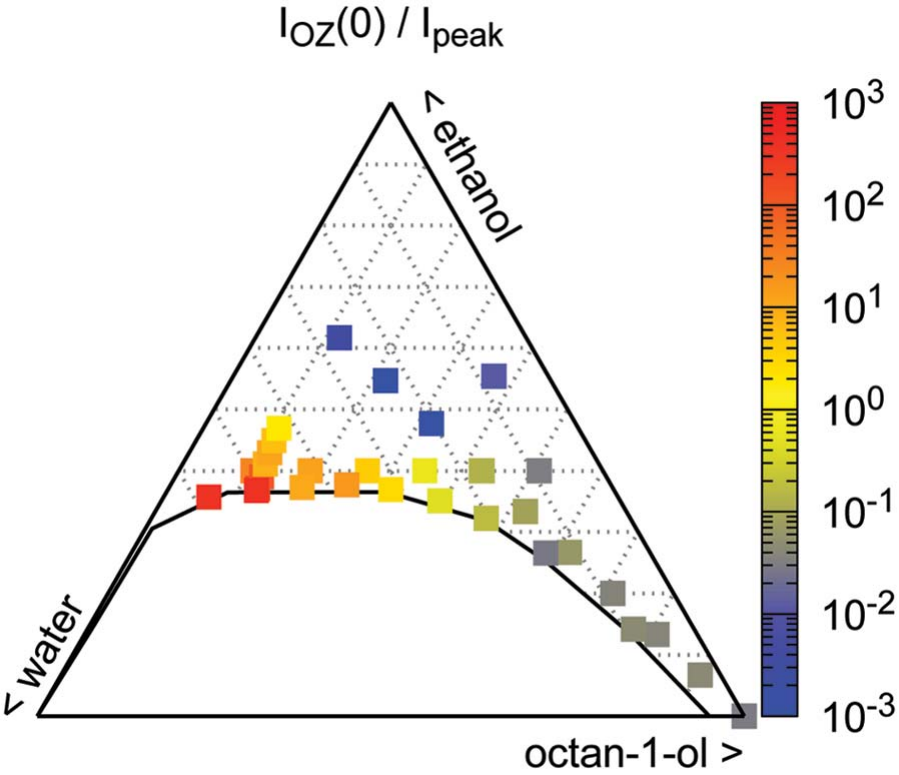
Ratios between the intensities of the OZ and broad-peak contributions to the model [equation (1)]. The two additive terms contribute very differently to the scattering observed, depending on the location in the phase diagram.

The fits to equation (1) and the two terms (shown as dashed lines) are presented in Fig. 3 ▸. Strikingly, the fits are able to capture all features of the experimental spectra in the three regions considered. As illustrated in Figs. 1 ▸(*b*)–1 ▸(*f*), the same model enables us to reproduce all the scattering patterns. The fit results of all spectra in Fig. 1 ▸ and the consequent interpretations are given in Figs. 5 ▸ and 6 ▸. The data are provided in Table S1 in the supporting information.

Interestingly, the broad-peak term not only is seen in the octan-1-ol-rich corner, but contributes significantly to the pattern even close to the critical point (see the water-rich spectrum in Fig. 3 ▸). This suggests that dense clusters of hydroxy groups still exist inside the direct pre-Ouzo aggregates.

When looking at the individual contributions, the first term of equation (1) (OZ term) is predominant when the composition of the sample lies in the water-rich part of the phase diagram. In contrast, on the oil-rich side, the second term (broad peak) provides the main contribution for fitting the experimental data. At intermediate water-to-octan-1-ol ratios, the terms contribute equally to the fit. As a consequence, the reliability of the fit parameters for the peak term on the water-rich side of the phase diagram decreases, while the same is true for the fit parameters from the OZ term on the water-poor side. In Fig. 5 ▸, the relative values of *I*_peak_ and *I*_OZ_(0) are plotted. As can be seen, this ratio spans over five orders of magnitude. We illustrate this by highlighting the predominance of either the first (red/orange squares in Fig. 5 ▸) or second term (blue/purple squares) using colours throughout the map.

As for conventional microemulsions, it is crucial to evaluate the fit quality by comparing the model and the data on both logarithmic and linear scales.

For samples in the range of 40–60% octan-1-ol and 30–40% ethanol, and near the phase transition, where the spectra are similar to those observed for bicontinuous microemulsions (Lopian *et al.*, 2016 ▸), we determine that the intensities of both contributions are of the same order of magnitude, as exemplified for the ‘bicontinuous’ spectrum of Fig. 3 ▸. Additionally, the mesh size from the broad peak and the correlation length from the fluctuations have similar values around 2 nm, which is also twice the length of an octan-1-ol chain. This overlap in dimensions may be the signature of a change of structure, and the need to swell beyond the maximum length of the alcohol seems to be the cause.

Scattering intensities scale with the number density *n*, the square average contrast 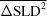 (SLD is the scattering length density) and the square average volume 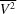 of the scatterers, the forward scattering being written for a single population of centrosymmetric aggregates in the monodisperse approximation as 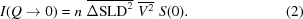
Here *S*(0) is the structure factor value at *Q* = 0 related to the isothermal compressibility, hereinafter considered as being equal to unity (no interaction considered). This equation gives access to another determination of the aggregate size, calculating a radius assuming spheres of square volume 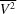. Since the basic property of a hydrotrope – here ethanol – is to distribute between phases and accumulate slightly near the interface, the estimation of SLDs to quantify the contrast is not straightforward. The procedure used is detailed in the supporting information.

**Figure 6:**
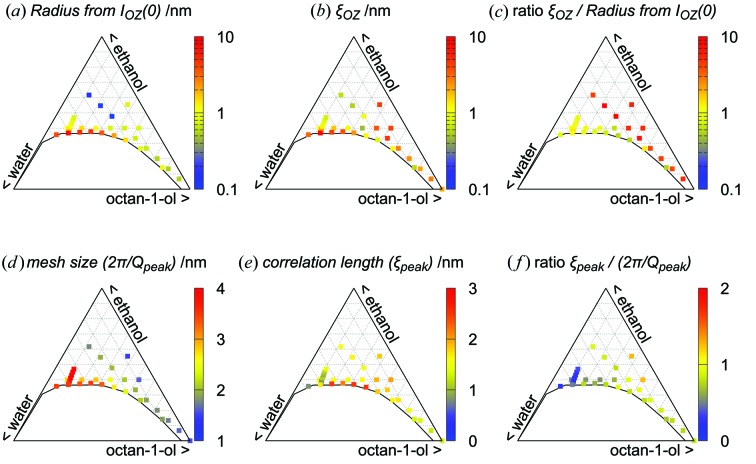
Fit outcomes for the combined Ornstein–Zernike fluctuations and Lorentzian (broad-peak) models [equation (1)].

We now turn to the general Fig. 6 ▸, which aims to assist the understanding of the main morphological structure variations within the wide monophasic domain investigated; additional plots are provided in the supporting information (Figs. S8 and S9). The ‘large’ OZ contribution is of course paramount in the vicinity of the critical point, located in the water-rich region. However, it extends in all directions and is still seen, for example, at 10% more hydrotrope than the critical point. The characteristic size of the aggregates, determined from the correlation length and from the forward scattering of this contribution, is in good agreement as long as the corresponding intensity is well above any ‘background’ level. This size ranges from slightly less than 1 nm up to 10 nm, reaching 10 nm at the binodal around the critical point, and decreasing in all directions from there. The self-consistency of the proposed model is demonstrated, as for classical micelles, by the equivalence of sizes as deduced from the forward intensity of the OZ contribution or from ξ_OZ_, which are in agreement, as shown in Figs. 6 ▸(*a*), 6 ▸(*b*) and 6 ▸(*c*).

The smaller distance, *i.e.* the mesh size of the reverse network from octan-1-ol (as shown in Fig. 4 ▸), determined from 2π/*Q*_peak_, extends from about 1 nm (similar to the octan-1-ol chain length) in the octan-1-ol corner to several nanometres (Fig. 6 ▸ *d*). The correlation length of this network, determined from the width of the peak (ξ_peak_), is a maximum close to the binodal and for intermediate compositions in octan-1-ol and water, *i.e.* in a region where bicontinuity is expected (Fig. 6 ▸ *e*). Fig. 6 ▸(*f*) shows that the ratio of distance to typical size, which is of the order of 3 for all classical microemulsions, is significantly lower here, *i.e.* between 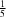 and 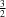. The UFME order does not propagate to the nearest-neighbour domains. This is consistent with the fact that UFMEs are stabilized not by surface film curvature but by a hydration force of much smaller decay length.

So far we have only presented SWAXS data. Note, however, that the small-angle neutron scattering (SANS) data are similar, as shown in the supporting information (Fig. S7) and by Diat *et al.* (2013 ▸). Two elements distinguish SANS and SAXS data for UFMEs. First, the contrast for SANS is created using partial deuteration, *e.g.* with fully deuterated octan-1-ol but hydrogenated ethanol and water. As a result, the OZ contribution to the scattering, corresponding to D-rich clusters in an H-rich medium, dominates. The peak contribution due to scattering by small hydroxy clusters in oil is thus often negligible, and water-rich spectra are well fitted with a simple OZ function. Second, with the sensitivity of neutrons to isotopic contrast, it is possible to use selective deuteration/hydrogenation to highlight the excess of hydrotrope (ethanol) which accumulates slightly at the interface; the corresponding scattering is weak and, in order to see it, other scattering contributions must be cancelled by matching the SLDs of the clusters and solvent. The existence of an interface of a particular SLD is neglected in our model for simplicity. Note that, as shown by Diat *et al.* (2013 ▸), the scattering by this excess of hydrotrope is also fitted by an OZ function, and would therefore be reproduced by our model. However, the simultaneous fit of different contrast conditions for the same morphology would show inconsistencies, *i.e.* the model would not fit all data if we were to change only the SLDs and keep the same correlation lengths.

## 4. Discussion   

The fits of experimental data by this simple model provide important insights into the behaviour and microstructure of these systems. However, the absolute scale is not used to constrain the fits, but exploited *a posteriori* to check the validity of the results. Also, this model does not include any description of a layer of hydrotropes at the surface of the direct pre-Ouzo aggregates. The use of equation (1) for fitting on both linear and logarithmic scales is different from the models used in other structured fluids. The long-standing OZ model, which is the first term in equation (1), is a Lorentzian (or Cauchy) function with its mode at zero that was created to reproduce the scattering from thermal fluctuations in composition with a correlation length ξ: 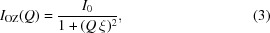
with an intensity that decays with a slope of *Q* ^−2^. Other non-particulate models with a plateau at low *Q* and no peak are also available in the literature, notably the Guinier approximation (Guinier & Fournet, 1955 ▸), 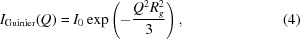
and the Debye–Anderson–Brumberger model (DAB) (Debye & Bueche, 1949 ▸; Debye *et al.*, 1957 ▸), which is simply the square of the OZ model: 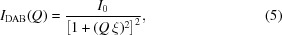
hence decaying as *Q* ^−4^. These models are shown in the supporting information (Fig. S2) but tend to be generally inadequate for the analysis of our data.

Non-particulate models that can reproduce a peak at *Q* 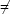 0 have also been proposed, in particular the broad-peak model, the spinodal decomposition model, the Teubner–Strey model and, interestingly, the generalized OZ model, also called the extended OZ model. The broad-peak model, which is the second term in equation (1), is often used for amorphous materials with a characteristic spacing of 2π/*Q*_peak_ and is a Lorentzian function with a nonzero mode:
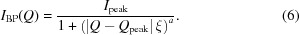
The high-*Q* regime follows *Q* ^−*a*^ [in equation (1) we fixed *a* = 2 as for the usual Lorentzian function]. The spinodal decomposition model due to Furukawa is
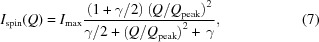
with the intensity at high *Q* decaying as *Q* ^−(2+γ)^, γ and *Q*_peak_ being related to the dimensionality and characteristic size of the structures in the unstable mixture. The Teubner–Strey model for bicontinuous microemulsions having similar volumes of both domains delimited by a sharp interface is (Teubner & Strey, 1987 ▸)
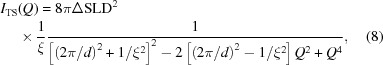
and can produce a peak if ξ > *d*/2π, *d* being the repetition distance of the domains; the high-*Q* intensity decays as *Q* ^−4^. These models (shown in Figs. S3 and S4 in the supporting information) can sometimes reproduce well those spectra exhibiting a strong peak, but will fail for water-rich samples. Recently, Bier & Harnau (2012 ▸) derived a model for fluids with ionic impurities, which extends the OZ formula by adding a term that depends on the charge of ions:
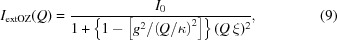
with 1/κ the Debye screening length and *g* an interaction contrast parameter. A peak might appear if, and only if, ions are present with a strong contrast between impurities and solvent, which the authors proposed can arise for a strong size asymmetry between the ion pair. This is related to the work of Onuki & Kitamura (2004 ▸) on solvation effects near critical points. Witala *et al.* (2016 ▸) successfully used this modified OZ function to account for a sharper plateau at low *Q* in the case of a binary liquid mixture with antagonistic salts. Note, however, that the ternary mixtures presented in our work do not contain ions beyond unavoidable traces, although the shape of the curves is sometimes strikingly similar. However, the modified OZ function cannot be used to fit all spectra, in particular in the oil-rich region.

There are some similarities between our model and the approach by Beaucage (1995 ▸) in aggregation, but without coexistence of immiscible liquids, which has the consequence of the existence of a smooth interface associated with a cutoff length (Arleth *et al.*, 2001 ▸). This case is opposite to the case of rigid microemulsions, when local curvature constraints induce the existence of a composition-dependent sharp peak in the scattering and also a Porod decay extending over more than one decade in intensity. For the calculation of the peak position in reduced units, the analytical predictive DOC model is available (Zemb, 1997 ▸). The Teubner–Strey expressions can be used, provided the flexibility of the film is close to 1*k*_B_ *T* per molecular area, while a model with two length scales has been used by Choi *et al.* (2002 ▸) for more rigid microemulsions.

Our model is able to fit the data presented here with one OZ function (a Lorentzian with its mode at zero wavevector), one Lorentzian nonzero mode and, when the WAXS domain is available, a series of Lorentzians with nonzero modes [see Figs. S6, and S5(*c*) and S5(*f*) in the supporting information]. Obviously, better fits could be achieved by adding more Lorentzian functions, with zero or nonzero modes. Whether these additional curves would occasionally have a physical meaning or not, such as capturing different existing fluctuations in size and/or SLD, is not yet known.

## 5. Conclusion   

In this work, we have described how to model all small-angle scattering patterns in UFMEs, based on a small physically defined number of structural parameters: basically one large correlation size due to the two immiscible fluids, and another contribution due to scattering from the internal structure of the solvent. After recognizing at least three different types of scattering pattern, we propose a unified fit model with a straightforward formula, which allows us to describe the scattering data in the complete monophasic region with the use of a very simple equation, based on just five parameters in addition to background.

The smaller length scale is due to the clustering of hydroxy groups in the octan-1-ol. Alcohols and other oils with a polar group that would create clusters are not necessary to obtain UFMEs from ternary mixtures of solvents, but are an obvious choice. The existence and the relative importance in scattering intensity of the Lorentzian contribution may vary greatly, depending on the system and the contrasts.

This model does not account for different actual structures in the system, as opposed to separate classical models for direct, inverse and bicontinuous microemulsions. It is, however, consistent with molecular dynamics simulations that can reproduce the main features of the scattering data (Lopian *et al.*, 2016 ▸) and show progressive growth of clusters when adding water to octan-1-ol/ethanol, up to and beyond percolation. In the particular case of UFMEs, highly polydisperse in nature, the domains overlap in the phase diagram and no well defined line of transition can be drawn: entropy, hydration forces (Parsegian & Zemb, 2011 ▸) and the structure of the oil-rich domains give different contributions to the free energy (Zemb *et al.*, 2016 ▸), leading to gradual changes. Rather, the important quantities to distinguish between different regions in the phase diagram are the size of the cluster (from the OZ contribution) and the spacing between reverse aggregates (from the broad-peak contribution).

We note that a better model will account for the excess of hydrotrope at the interface between clusters and solvent, to be able notably to reproduce SAS data with particular contrasts, *e.g.* SANS with hydrotrope contrast as exemplified by Diat *et al.* (2013 ▸). Simultaneously, the absolute scale should be included by accounting for pseudo-partition coefficients between the two domains (and the interface), while this also forces us to consider the thermodynamic consistency of the fit parameters.

It is interesting to note that we find for UFMEs the same three morphologies known for both classical and rigid microemulsions: direct droplets, connected reverse droplets, and an intermediate case similar to bicontinuous micro­emulsions (Lopian *et al.*, 2016 ▸). Whether locally lamellar or bi-liquid foams or sponge phases also exist in UFMEs is still an open question in our opinion.

## Supplementary Material

Additional figures and table. DOI: 10.1107/S1600576716016150/zg5005sup1.pdf
