# Supplementary material for: Small-angle scattering and morphologies of ultra-flexible microemulsions
Source: J Appl Crystallogr. 2016 Nov 11;49(Pt 6):2063–72. doi: 10.1107/S1600576716016150 (PMC5139994; doi:10.1107/S1600576716016150)
Supplement: Supplementary file 1 [file j-49-02063-sup1.pdf]

Small-Angle Scattering and morphologies of ultra-flexible microemulsions

Supplementary Materials

Sylvain Prévost[1*], Tobias Lopian[2,3], Maximilian Pleines[2,3], Olivier Diat[2], Thomas Zemb[2]

1- ESRF – The European synchrotron, 71 avenue des Martyrs, 38000 Grenoble, France
2- Institut de Chimie Séparative de Marcoule (ICSM), UMR 5257 (CEA/CNRS/UM2/ENCSM), 30207 Bagnols-sur-Cèze, France
3- Institute of Physical and Theoretical Chemistry, University of Regensburg, 93040 Regensburg, Germany

* Corresponding authors: sylvain.prevost@esrf.fr, thomas.zemb@icsm.fr

Table of content

- Figure S1: ternary phase diagram of octan-1-ol/ethanol/water in (a) mass fraction, (b) volume fraction – taking the densities of pure solvents, and (c) mole fraction.
- Estimate of the contrasts in the direct and reverse domains.
- Table S1: Best fit parameters for spectra presented Fig. 1 in the main article.
- Figure S2: fit of SAXS spectra from water-rich samples by only Ornstein-Zernicke function, and in one case with the DAB and Guinier model.
- Figure S3: fit of SAXS spectra from samples at intermediate compositions in water and octan-1-ol, with the Teubner-Strey model and the extended Ornstein-Zernicke model.
- Figure S4: fit of SAXS spectra from octanol-rich samples by Hard Spheres.
- Figure S5: Individual and summed contributions of the model for 3 spectra away from the binodal, in log-log and lin-lin scales.
- Figure S6: Example of the use of a non-constant background, fitting the WAXS peaks by a sum of Lorentzian whose tails at low Q replace the constant background otherwise used in the SAXS Q-range.
- Figure S7: Fits of SANS data
- Figure S8: curves from parameters in Table S1, demonstration of the dominant contributions
- Figure S9: curves from parameters in Table S1

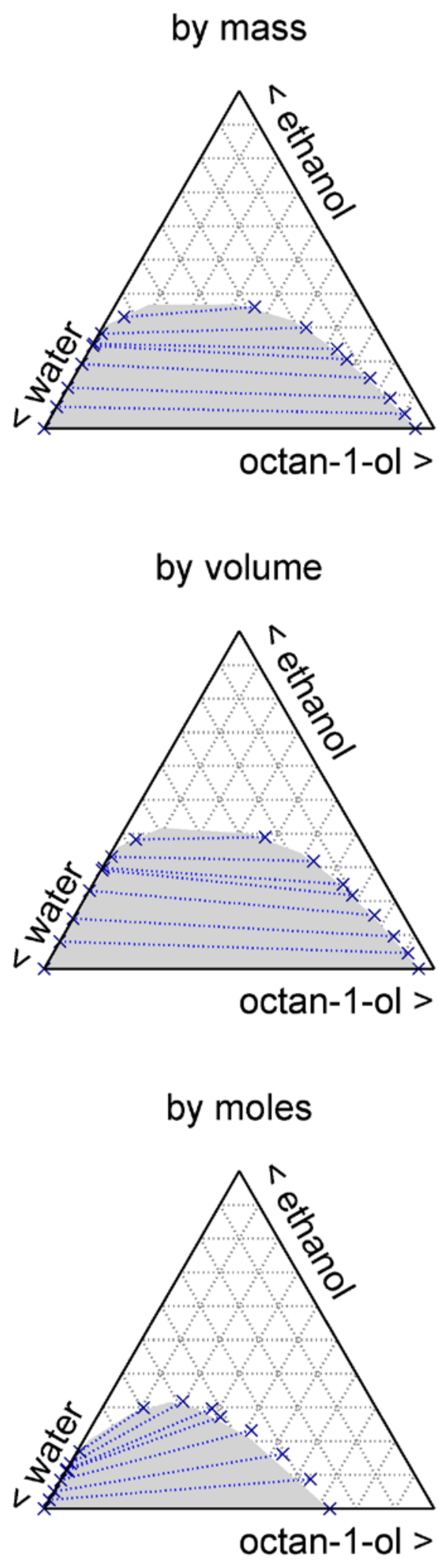

Figure S1: ternary phase diagram of octan-1-ol/ethanol/water in (a) mass fraction, (b) volume fraction – taking the densities of pure solvents, and (c) mole fraction. The grey area was determined in our lab, the blue points are from (Arce *et al.*, 1994). A small discrepancy appears in mole fractions, but the shape of the biphasic domain remains.

Estimate of the contrasts in the water-rich and octan-1-ol-rich regions

The contrast can be estimated, though three difficulties arise in the particular case investigated here: (i) The contrast and the number density both require to know accurately the actual composition of the domains in the liquid, while we do not know exactly how the 3 components octan-1-ol/ethanol/water spatially separate into domains for the OZ contribution, or how hydroxy groups and water molecules segregate from an aliphatic-rich matrix for the broad peak contribution, (ii) contrast and volume both require to know the actual partial molar volume of each compound on the different domains, and finally and importantly, we are here considering domains of very similar electronic densities, as based on C, H and O atoms, thus the X-ray contrast is little, and approximations over scattering length densities will propagate and amplify when calculating the square of the contrast.

As a simplification, we can assume that partial molar volumes are constant, a fact supported by density data on this ternary system by Arce et al. (Arce *et al.*, 1993); the main deviation to ideal mixture is observed for the binary water/ethanol with only a 4 % change in density. Using densities of pure liquids (0.8217 g·cm^{-3} for octan-1-ol, 0.7851 for ethanol, 0.9970 for water), we find X-rays coherent scattering length densities of respectively 7.93E-4 nm^{-2}, 7.57E-4 and 9.41E-4. From molecular dynamics and contrast variation SANS, we also know that, near the critical point, ethanol is essentially shared equally between the oil-rich and the water-rich domains, i.e. the same distribution as for the two phases in equilibrium on the binodal (Arce *et al.*, 1994). As the miscibility gap is closing for ca. 40 % ethanol, we can consider a composition of 50 % ethanol for many samples presented in the current work. The contrast between 1/1 octan-1-ol/ethanol and 1/1 water/ethanol domains is conveniently of 1E-4 nm^{-2}. Noting that $n\langle V\rangle$ (number density times average volume) is the volume fraction, and neglecting interactions, Eq. 2 of the main article leads to $I/nm^{-1} \approx 10^{-8}\ \phi V$. This (rough) approximation helps validating the dimensional parameter ξ_{OZ} independently. For example, droplets of volume 1000 nm³ (correlation length around 10 nm) at a volume fraction of 10 % would lead to a forward scattering of 10^{-6} nm^{-1}, or 1 mm^{-1}. In the octan-1-ol/ethanol/water system such intensity is only reached or exceeded for samples on the binodal near the critical point where the correlation length of domains is found to be around 10 nm, which is in good agreement with the 1000 nm³ volume size. Away from the binodal and the critical point, the volume fraction of <<10 % of such droplets is a reasonable estimation, which *a posteriori* may justify neglecting interactions.

For the oil-rich domain, dense clusters of hydrated hydroxyls produce the correlation peak. From the densities of linear alcohols, the volume of a hydroxyl is estimated to be approximately 0.014 nm³, with a scattering length density of 18E-4 nm^{-1} and a volume of the octan-1-ol aliphatic chain 0.248 nm³, giving a scattering length density of 7.4E-4 nm^{-1}. Thus, the resulting contrast is 10.6E-4 nm^{-1}. By adding water, which possesses a molecular volume of 0.030 nm³, this contrast decreases drastically, e.g. considering 3 water molecules per hydroxyl the contrast goes down to 3E-4 nm^{-1}. This loss in contrast will result in a 10-fold decrease in intensity.

Weight fractions			Fit parameters				
Octan-1-ol	Ethanol	Water	ξ_{OZ} /nm	$I_{OZ}(0)$ /mm^{-1}	ξ_{peak} /nm	$2\pi/Q_{peak}$ /nm	I_{peak} /mm^{-1}
Fig. 1 b							
1.0000	0.0000	0.0000	2.00	6.70E-04	1.26	4.05	2.35E-02
0.9041	0.0669	0.0290	2.74	9.66E-04	1.21	3.84	2.30E-02
0.8098	0.1331	0.0571	2.10	9.38E-04	1.33	3.63	2.59E-02
0.7140	0.2000	0.0860	2.43	1.01E-03	1.50	3.51	2.80E-02
0.6190	0.2670	0.1140	2.70	1.81E-03	1.70	3.37	2.87E-02
0.5235	0.3337	0.1429	4.69	2.13E-03	1.82	3.24	2.58E-02
0.4280	0.4000	0.1720	3.57	2.64E-03	1.91	3.10	2.11E-02
0.3190	0.4770	0.2040	0.99	3.77E-06	1.55	3.17	1.17E-02
0.2190	0.5470	0.2340	0.53	1.06E-07	1.63	3.25	8.20E-03
0.1190	0.6170	0.2640	0.59	2.22E-05	1.57	3.54	6.73E-03
Fig. 1 c							
0.1260	0.3710	0.5030	4.40	2.78E-01	1.11	1.96	1.65E-03
0.1220	0.3900	0.4880	2.08	6.80E-02	0.79	2.26	1.20E-03
0.1170	0.4110	0.4720	0.95	2.07E-02	1.08	2.99	8.42E-04
0.1139	0.4296	0.4565	0.90	1.76E-02	0.96	2.70	9.02E-04
0.1099	0.4496	0.4406	0.70	1.16E-02	1.09	2.54	7.83E-04
0.1069	0.4695	0.4236	0.49	8.61E-03	1.42	4.55	3.82E-04
Fig. 1 d							
0.1050	0.4000	0.4950	1.21	2.53E-02	1.39	2.24	9.42E-04
0.1860	0.4000	0.4140	1.69	4.81E-02	1.54	2.20	4.01E-03
0.2670	0.4000	0.3330	1.19	2.36E-02	1.52	2.20	6.00E-03
0.3480	0.4000	0.2520	0.45	4.88E-03	1.60	2.50	8.16E-03
0.4290	0.4000	0.1710	3.57	2.64E-03	1.91	3.10	2.11E-02
0.5100	0.4000	0.0900	4.18	8.54E-04	2.00	3.91	2.82E-02
Fig. 1 e							
0.0640	0.3570	0.5790	3.19	9.85E-01	0.78	1.80	2.99E-03
0.1260	0.3680	0.5060	10.11	1.23E+01	1.69	1.80	3.50E-02
0.1880	0.3730	0.4390	4.26	2.10E+00	2.50	1.89	2.06E-01
0.2490	0.3770	0.3740	3.89	1.99E+00	2.16	1.80	1.21E-01
0.3153	0.3694	0.3153	2.33	6.22E-01	2.50	1.89	2.27E-01
0.3940	0.3510	0.2550	1.25	1.45E-01	2.50	2.34	3.29E-01
0.4735	0.3227	0.2038	0.80	3.53E-02	1.84	2.67	2.22E-01
0.5864	0.2657	0.1479	1.15	7.05E-03	1.62	3.01	2.65E-01
0.7722	0.1419	0.0859	0.58	1.31E-02	1.30	3.39	3.02E-01
Fig. 1 f							
0.3690	0.5540	0.0770	4.46	2.13E-04	1.84	4.33	1.92E-02
0.3190	0.4770	0.2040	0.99	3.77E-06	1.55	3.17	1.17E-02
0.2670	0.4000	0.3330	1.19	2.36E-02	1.52	2.20	6.00E-03

Table S1: Compositions and best fit parameters for spectra presented Fig. 1 in the main article.

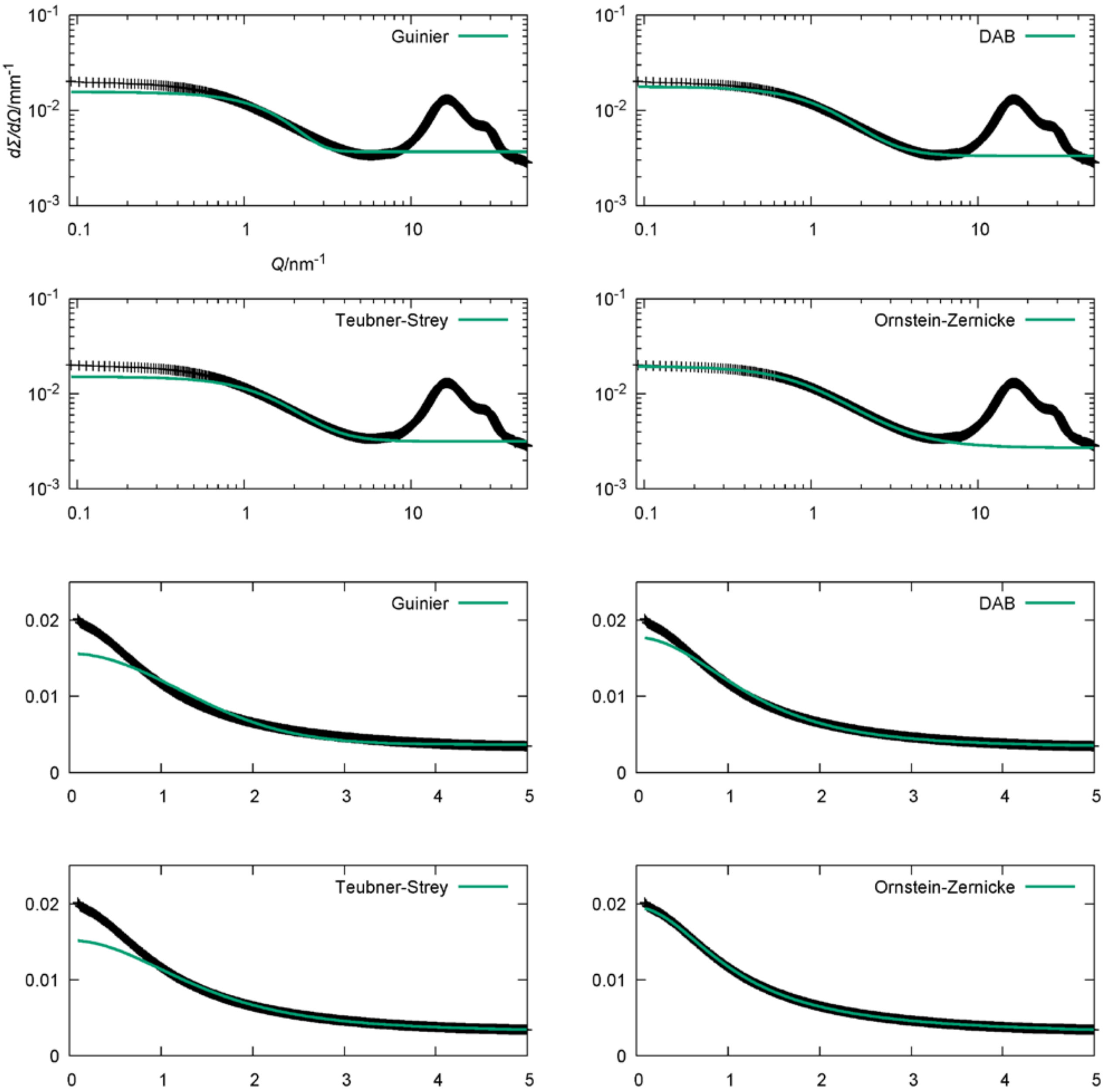

Figure S2: Comparison of a SAXS spectrum from a water-rich sample (octan-1-ol/ethanol/water 11/43/46) with Guinier, DAB, Teubner-Strey and Ornstein-Zernicke models, in log-log then lin-lin scales. The fits are performed with a constant background on the Q range from 0.1 to 5.8 nm^{-1}. All models reproduce the main features of spectra in this water-rich side of the phase diagram (plateau and smooth intensity decay). The Guinier model is the most inappropriate, as it decays too quickly at high Q. The Teubner-Strey has a similar problem, causing the plateau to be poorly reproduced in order to fit better the decay. The DAB model fits quite well, though it would not reproduce the peak seen at other compositions. The extended Ornstein-Zernicke is not represented here, as its parameters can be adjusted to result in the same function as the regular Ornstein-Zernicke.

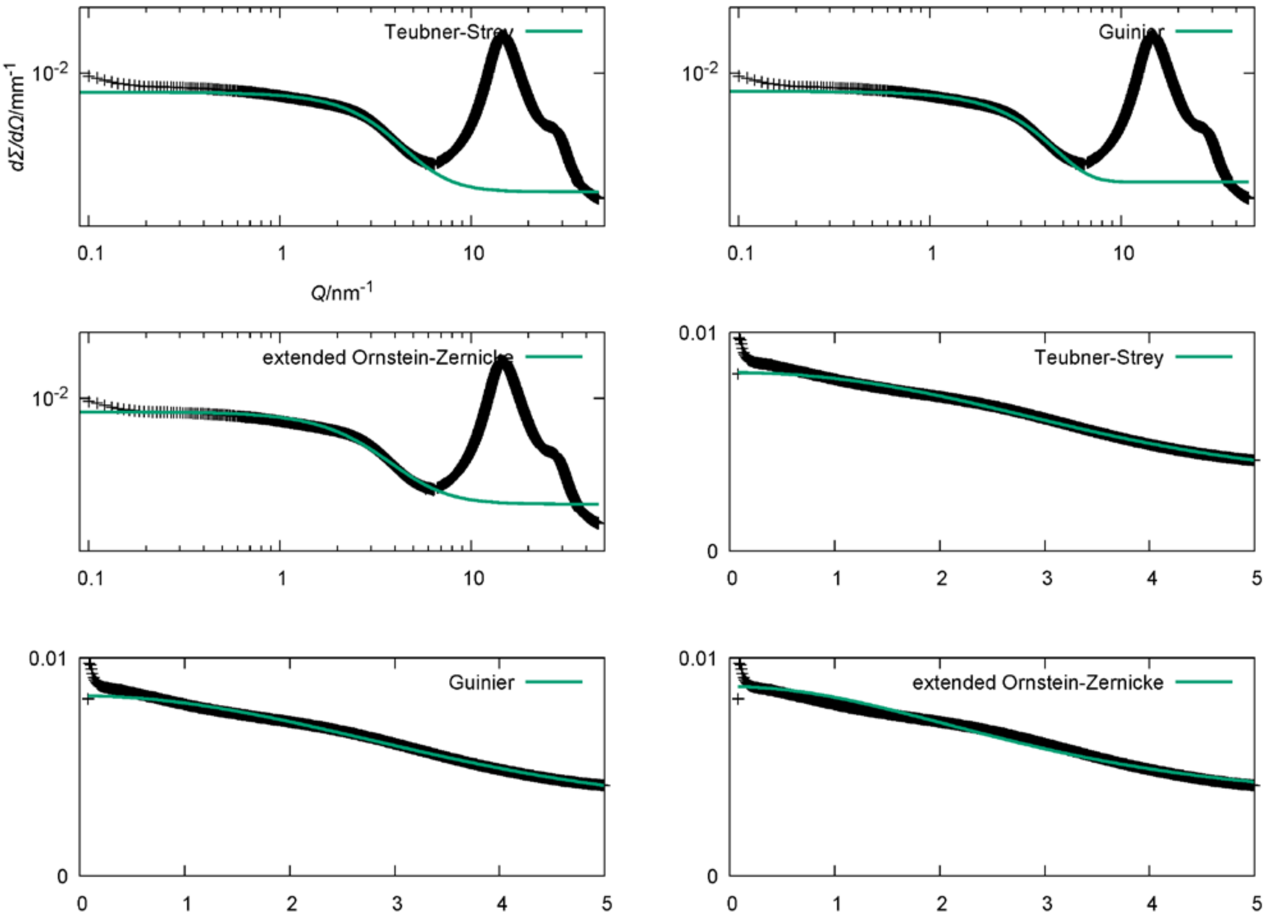

Figure S3: Comparison of a SAXS spectrum from a sample at intermediate compositions (octan-1-ol/ethanol/water 43/40/17), with the Teubner-Strey, Guinier, and the extended Ornstein-Zernicke models, in log-log then lin-lin scales. The fits are performed with a constant background on the Q range from 0.1 to 5.8 nm^{-1}. While overall in agreement with the data, these models fail to capture in detail the two features of the experimental data: plateau at low Q and kink at mid Q. The DAB and OZ models are not represented, as thez cannot reproduce a peak or kink and have an even smoother decay than Guinier.

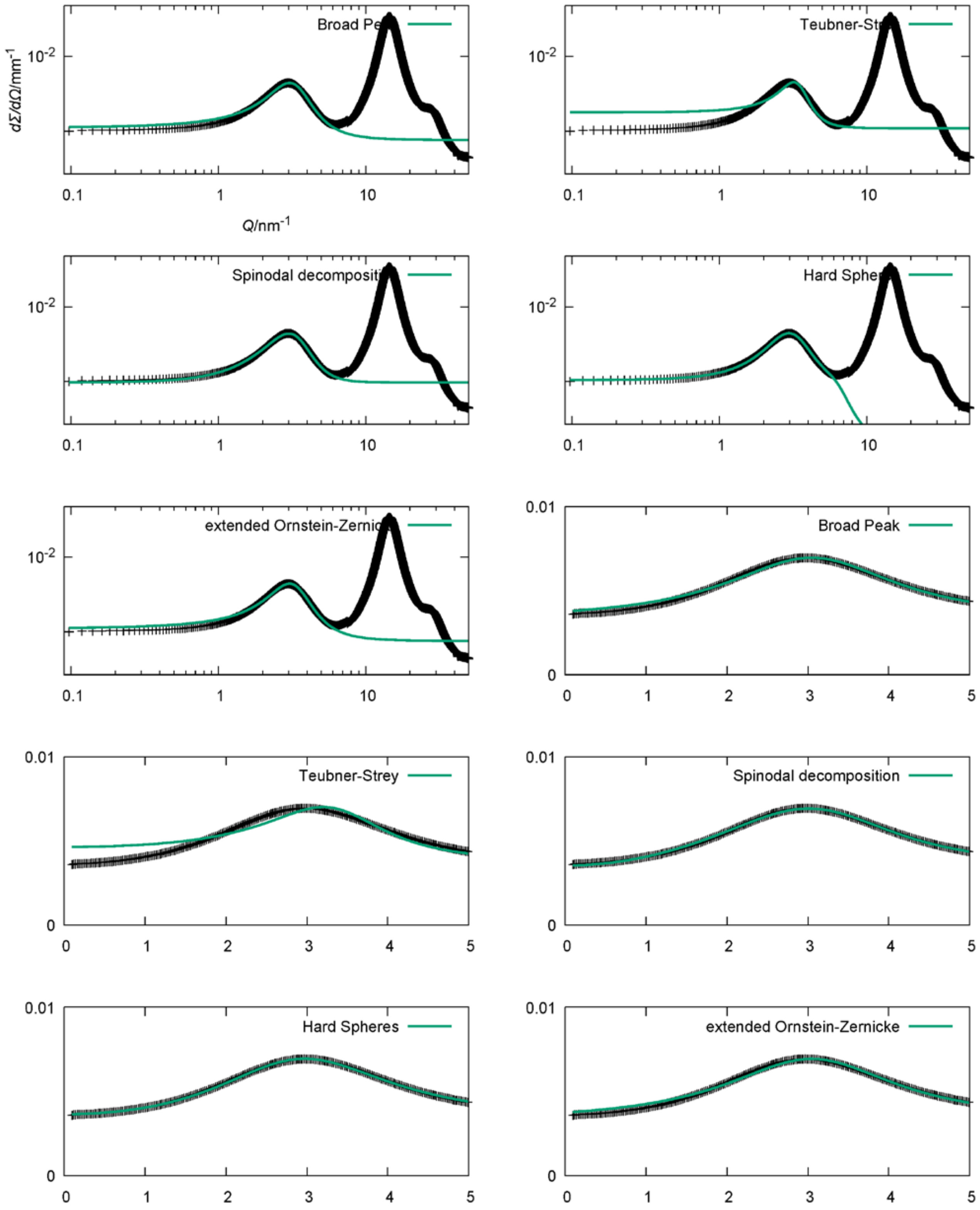

Figure S4: Comparison of a SAXS spectrum from an octanol-rich sample (octan-1-ol/ethanol/water 59/26/15) with Broad Peak, Teubner-Strey, Spinodal decomposition, Hard Spheres (spheres form factor with hard sphere structure factor) and extended Ornstein-Zernicke models, in log-log then lin-lin scales. The fits are performed with a constant background on the Q range from 0.1 to 5.8 nm^{-1}. Except the extended Ornstein-Zernicke, models can reproduce well the data, but would fail at reproducing the water-rich spectra.

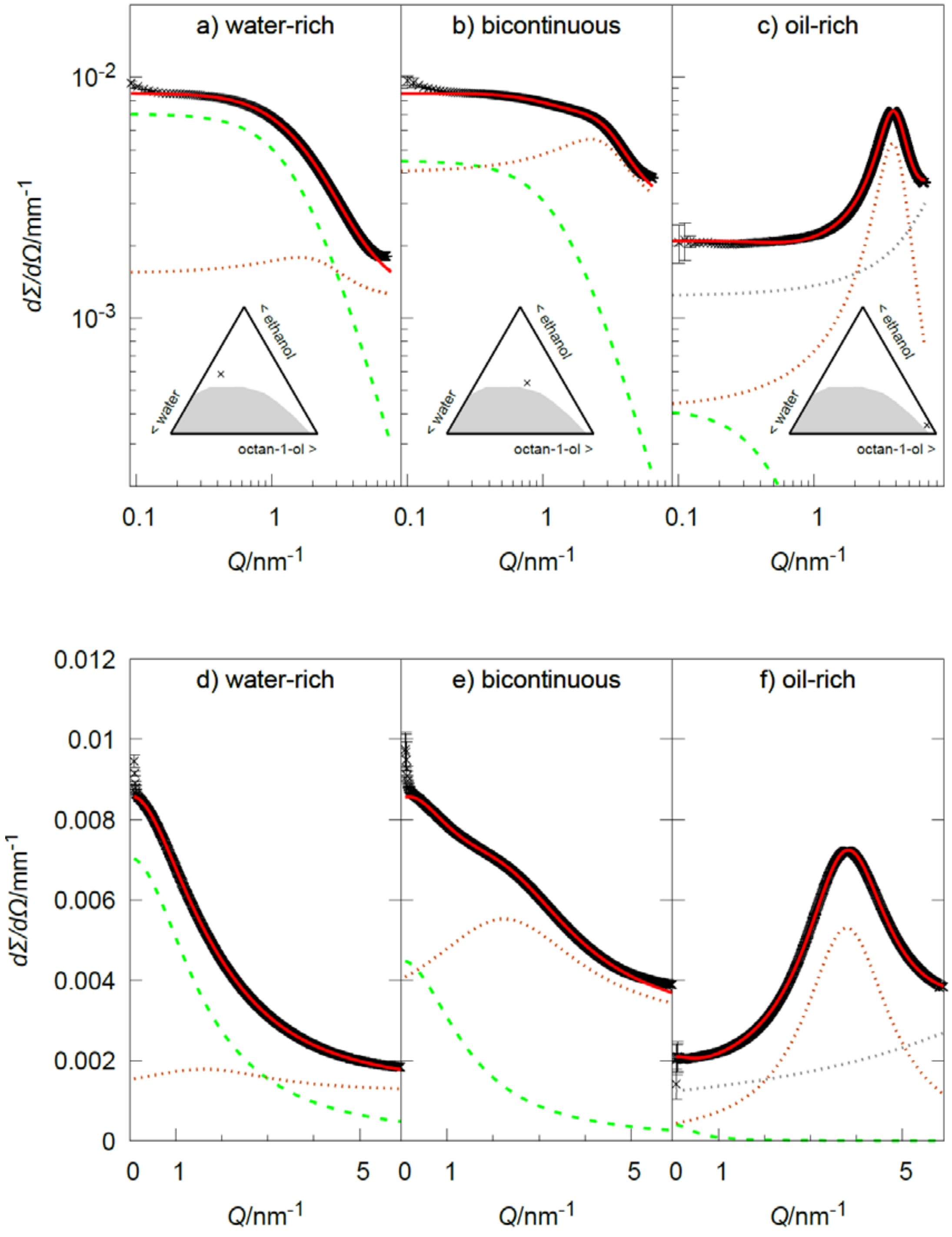

Figure S5: Individual and summed contributions of the model for 3 spectra away from the binodal, in double logarithmic scale (a, b and c) and double linear scale (d, e and f). The mass ratios of octan-1-ol, ethanol and water are respectively 0.11/0.45/0.44, 0.35/0.40/0.25 and 0.90/0.07/0.03. Red continuous lines are fits by Eq. 1, and the green and orange dashed lines are the OZ and Lorentzian terms. The last sample near pure octan-1-ol exemplify the importance of using a non-constant background in some cases, replacing the constant by a sum of Lorentzian fitting the WAXS range, see Fig. S6. This non-constant background is displayed as a dashed grey line in c and f.

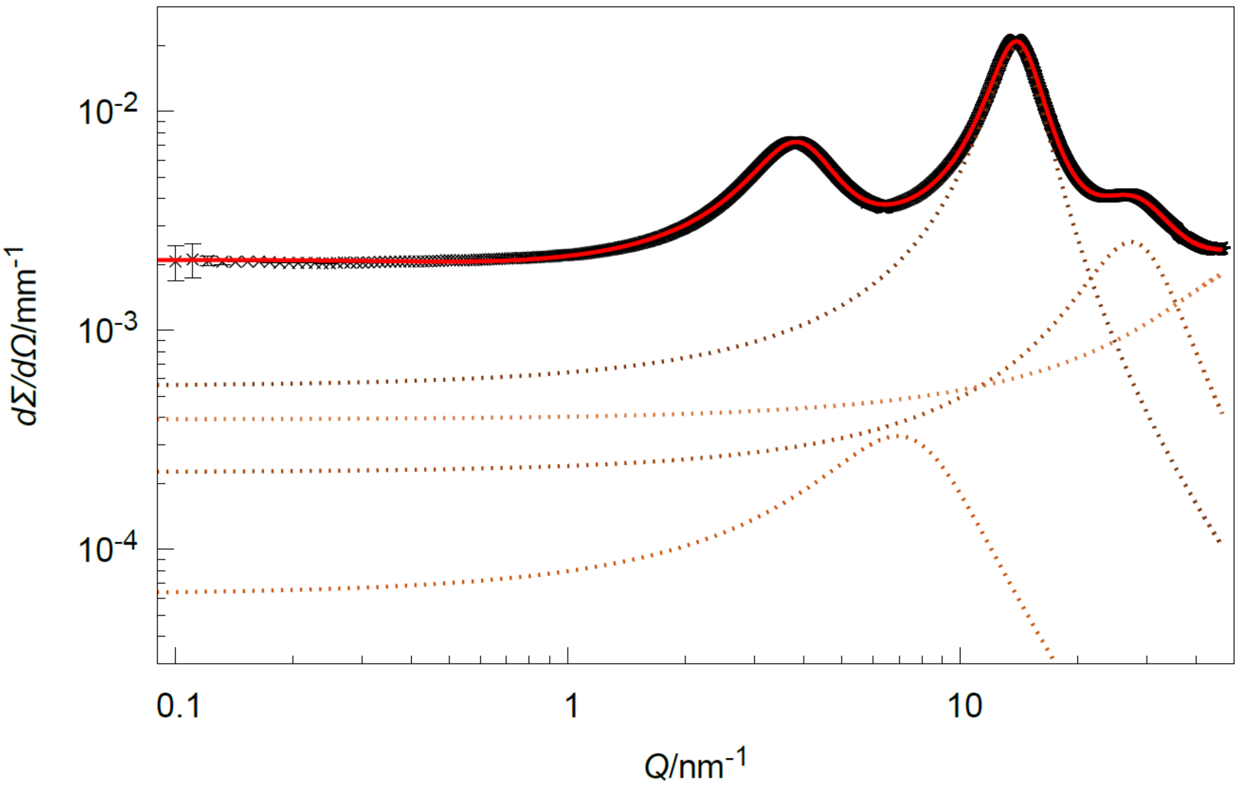

Figure S6: Background estimated as a sum of 4 Lorentzian functions fitting the inter- and intra-molecular peaks in the WAXS regime. The sum of these Lorentzians is the grey curve in Fig. S5 (c) and (f). In most cases, the SAXS Q-range can be fitted assuming a constant background instead; in the octan-1-ol corner however, the proximity of the mid Q peak to WAXS signal requires to determine a more precise background that accounts better for molecular scattering.

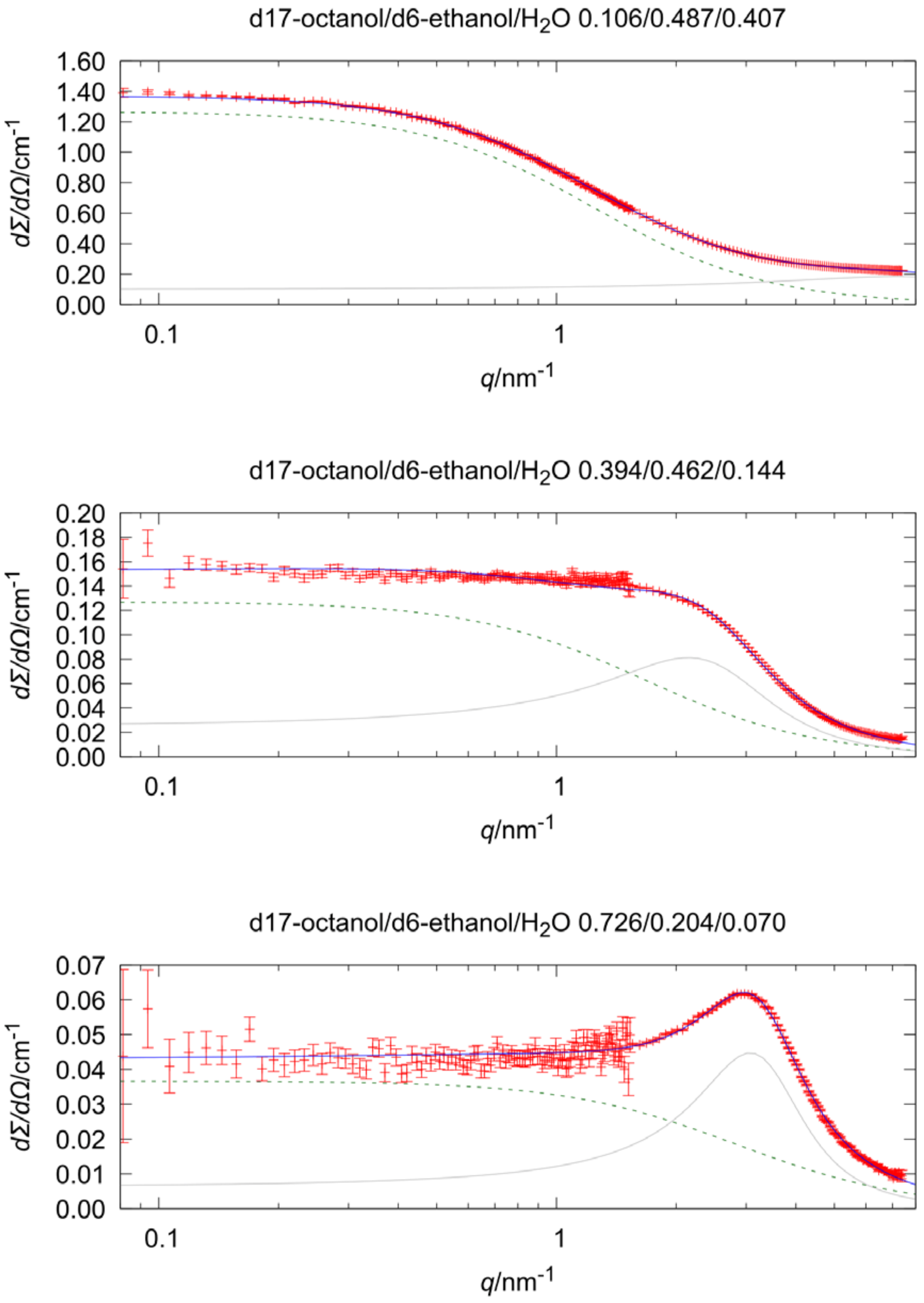

Figure S7: SANS data from D22, ILL, Grenoble, France (experimental data in red with constant background subtracted) and fit with Eq. 1 (blue line). The Ornstein-Zernicke signal is plotted as a green dashed curve, the Lorentzian is plotted as a grey dotted line. Compositions are given in volume fractions.

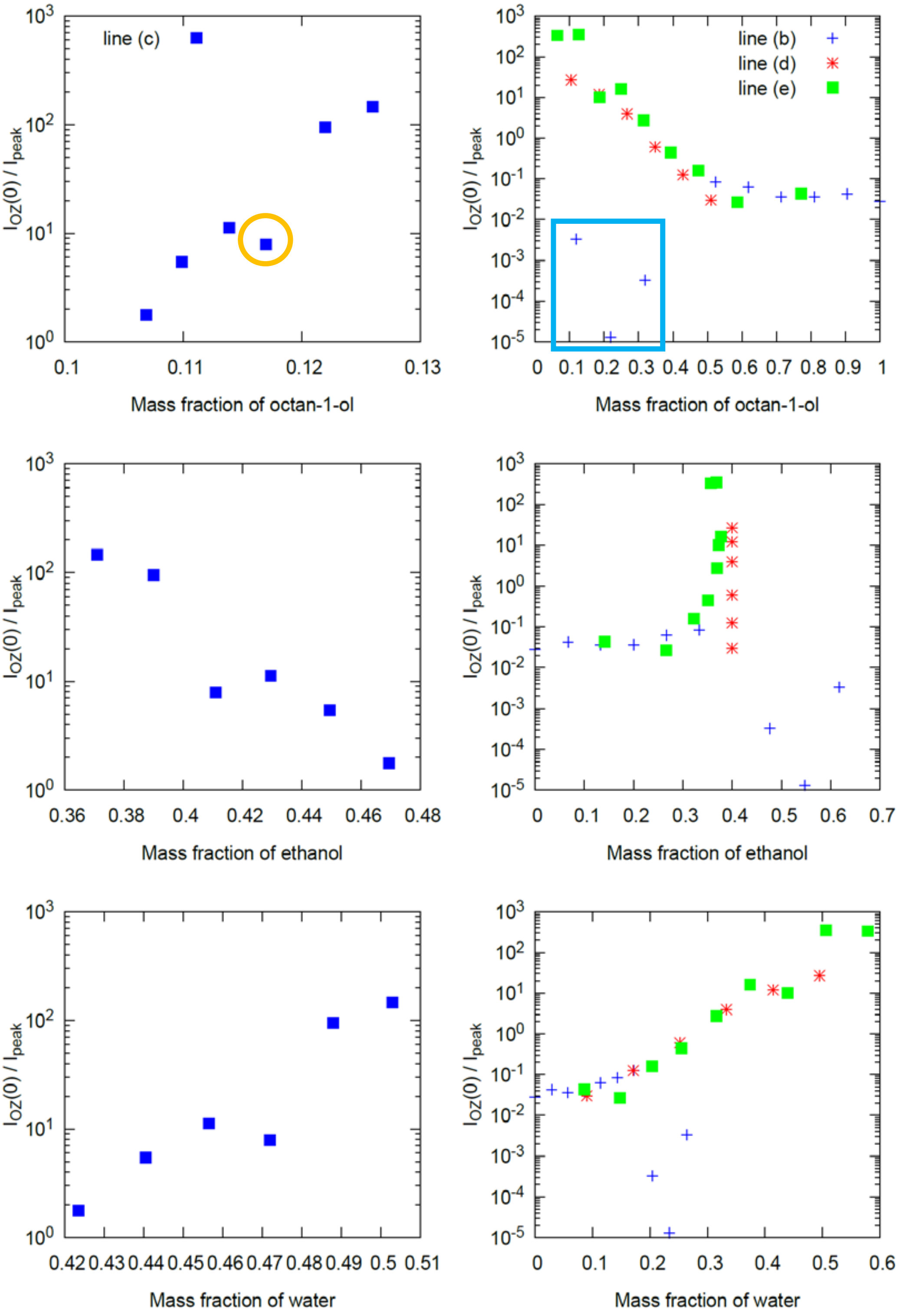

Figure S8: Data from Table S1, corresponding to Figure 5 in the main article, for 4 dilution lines (b, c, d, and e, see Figure 1). The ratio between the two contributions in Eq. 1 varies greatly depending on the composition. In particular, the Ornstein-Zernicke contribution vanishes in the ethanol corner, when the ternary system becomes a molecular fluid. This explains why, for the dilution line b, points in the blue rectangle seem randomly distributed between 1E-5 and 1E-3, as we have reached the experimental precision. For dilution line c, the point highlighted by an orange circle is an outlier, maybe due to a small mismatch in sample composition; fit results were reproducible.

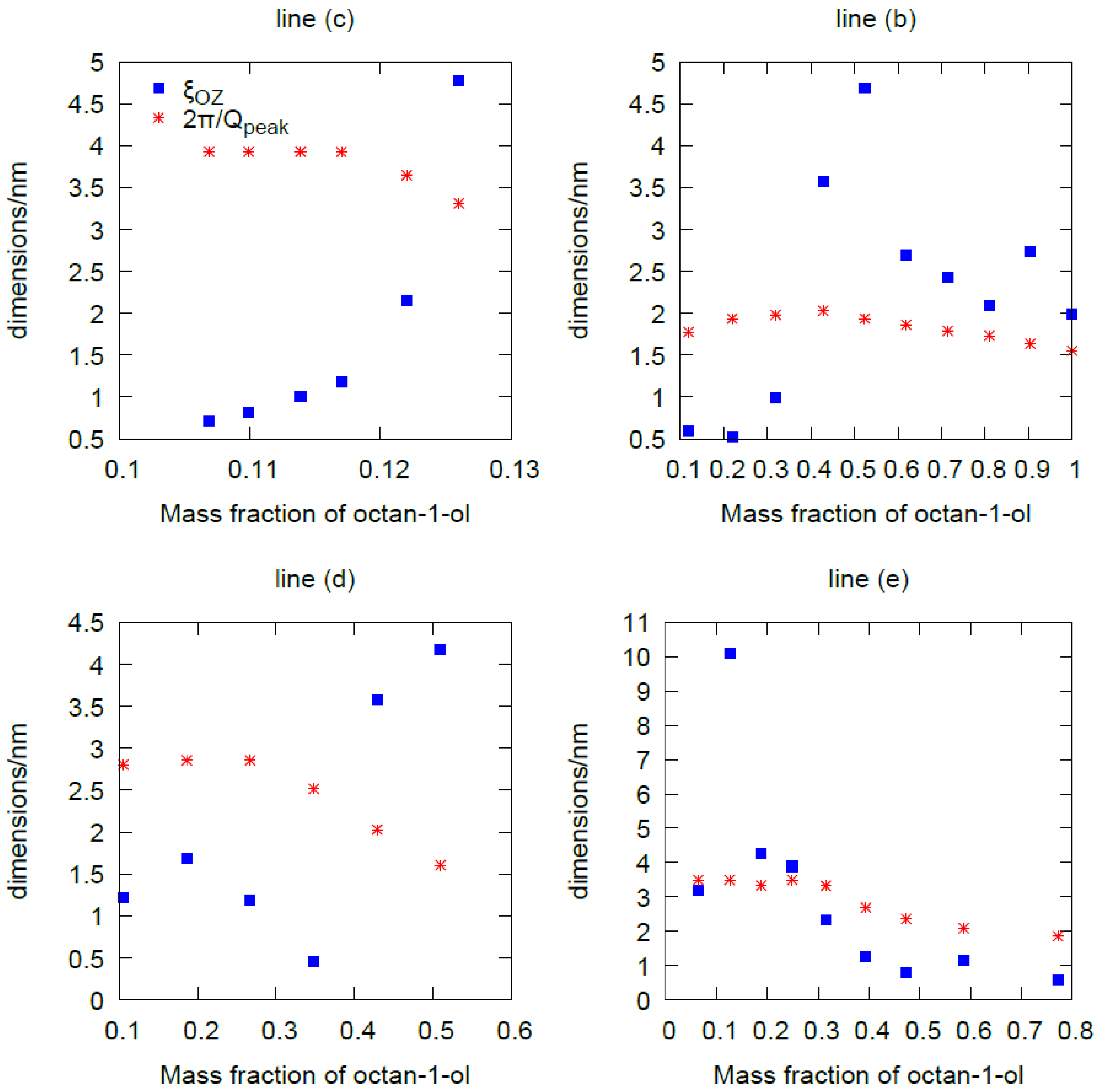

Figure S9: characteristic distances of both contributions, ξ_{OZ} and $2\pi/Q_{peak}$, for the dilution lines b, c, d and e in Figure 1. $2\pi/Q_{peak}$ is due to the distance between hydroxyl groups and is always larger than the length of the octan-1-ol molecule. It also does not extend much beyond ca. two octan-1-ol molecules. ξ_{OZ} varies much more, from a molecular size (ca. 0.5 nm) to ca. 10 nm.

References:

Arce, A., Blanco, A., Soto, A. & Vidal, I. (1993). *J. Chem. Eng. Data*. **38**, 336–340.

Arce, A., Blanco, A., Souza, P. & Vidal, I. (1994). *J. Chem. Eng. Data*. **39**, 378–380.
